# The Organizational Commitment in the Company and Its Relationship With the Psychological Contract

**DOI:** 10.3389/fpsyg.2020.609211

**Published:** 2021-01-14

**Authors:** Juan Herrera, Carlos De Las Heras-Rosas

**Affiliations:** Department of Economics and Business Administration, Universidad de Málaga, Málaga, Spain

**Keywords:** psychological contract, organizational commitment, HR management, job insecurity, bibliometric, SciMAT

## Abstract

Business organizations in their work environment, aspire to create a high level of performance and low levels of absenteeism and turnover. Organizational commitment is considered a key factor in achieving this objective, however, it can be conditioned by several factors, among which is the psychological contract. The literature has related the organizational commitment with the fulfillment of the psychological contract framing it as one of the explanatory variables. This work aims to investigate research trends on psychological contract and organizational commitment. For this purpose, bibliometric techniques and the software SciMAT have been used. 220 journal articles indexed in Web of Science (WoS) were analyzed. The findings indicate that the theme chosen for this review is valid. Based on the relationship between the two concepts, as the most recurrent themes, issues such as the sense of justice and the consequences of the violation of the psychological contract, normative commitment, HR management or job insecurity are addressed. However, in the last period analyzed (2015–2018), publications related to more sensitive topics to the present time emerge, such as the employability or the impact of these two concepts in the new generations (millennial and generation-Y) or the retention of talent. On the other hand, shortcomings are detected in the research on the ideologically charged psychological contract, the analysis of the organizational context or cultural and demographic factors in relation to both theoretical constructs. The contribution of this work lies in giving visibility to scientific results, which will serve business organizations as instruments for decision making in their labor management and, for the scientific community, as knowledge of the research spaces to explore.

## Introduction

In the current business context, a change toward an efficiency model based on organizational commitment is required. Business organizations need to form teams that are highly committed to their strategic objectives, oriented toward organization and work. Human resource management is positioned as one of the main functions within the organization where working conditions, worker welfare and job satisfaction are valued, which helps to maintain high levels of organizational commitment (Tiwari and Singh, 2014; Kurtessis et al., 2017). The formation of organizational commitment is related to the inputs that the worker receives from the organization and is intimately linked to the results of the relationship between both parties, as well as to the emotional bond between the goals and values of the organization and the employee (Buchanan, 1974). This exchange relationship between worker and company can affect work performance, absenteeism and job rotation (Betanzos and Paz, 2007). The literature in many works has related the organizational commitment to the fulfillment of the psychological contract, that is, the degree of compliance with the promises made by the organization (Rousseau and Parks, 1993), framing it as an explanatory and determining variable of the organizational commitment (Guest, 1998; Zaragoza and Solanes Puchol, 2013). The following provides a review of these two concepts; psychological contract and organizational commitment, as well as the relationship between them.

### Psychological Contract

Rousseau (1995) defined the psychological contract as the set of individual beliefs of a person in relation to the reciprocal obligations and benefits established in a relationship of exchange. The result of the exchange conditions the behavior of both the organization and the employees. However, Rousseau emphasizes in her definition the unipersonal and subjective nature of the employees’ interpretation of the psychological contract, which could lead to different views on the terms of the psychological contract between employer and employee (Morrison and Robinson, 1997).

The theory of the psychological contract shows as a key factor the perceived adequate performance of the contract between two parties involved, where subjectively one of the parties considers that the other has not adequately performed the obligations promised (Robinson, 1996). In reality, this comparison has consequences that go beyond the mere violation of the expected rewards. Its influence extends to the image that the person acquires about the organization, affects trust in the employer and the perception of fairness in the employment relationship (Cantisano et al., 2008).

In the exchange relationship between the organization and the employee two main dimensions can be distinguished, the relational contracts and the transactional contracts (MacNeil, 1985). The relational are characterized by an emotional exchange of social-emotional resources in the long term, in this case, the employee perceives that in exchange for his loyalty he will receive possible promotions to develop his career in the organization and/or security to remain in his job. The transactional contracts are mainly focused on an economic exchange (Rousseau, 1995). The breach of the psychological contract and its consequences have been widely addressed by researchers, where it has been demonstrated in many empirical studies how this fact is positioned as a key aspect in the field of labor relations and influences negatively the attitudes and behavior of employees (Coyle-Shapiro et al., 2019). The validity or degree of compliance with the psychological contract has different interpretations depending on whether the employee is involved (Robinson, 1996), the employer (Coyle-Shapiro and Kessler, 2002) or both (Dabos and Rousseau, 2004). A distinction must be made between breach and violation of the psychological contract. While the assessment of compliance or breach of the psychological contract starts from the individual’s own analysis based on his or her judgment, the violation of the psychological contract is a possible consequence of the breach, where the subject reacts in an emotional way, with greater resentment and indignation (Coyle-Shapiro et al., 2019).

The consequences associated with the breach of the psychological contract can be numerous and depend on a variety of factors that would be related to both the organization and the employee. Some of these consequences have been widely addressed by researchers; employees’ job performance (Gracia et al., 2006); intention to leave work (Lester et al., 2002); job satisfaction and organizational commitment (Porter et al., 1998; Cassar, 2001; Coyle-Shapiro and Kessler, 2002; Topa and Morales, 2005); and/or decreased trust (Robinson, 1996; Zhao et al., 2007), among others. The difference between the promises made and their fulfillment will generate different effects depending on the characteristics of the labor relationship, the age of the employee, the professional category, the organizational culture (individualistic or collective) and other demographic variables (Rousseau, 1995; Turnley and Feldman, 1999; Costa et al., 2017). As for the emerging and developing themes on the psychological contract, Coyle-Shapiro et al. (2019) point out that they focus mainly on (a) those who investigate an expansion of psychological contracts beyond social exchange, the so-called psychological contracts with a strong ideological charge and their relation to organizational commitment, the presence of this type of contract in the public sector, or its repercussion on the organizational citizen behavior of employees, (b) those who analyze the antecedents of the breach of the psychological contract, such as the restructuring and downsizing of organizations, causing a decrease in the capacity to fulfill their part of the exchange, or those who investigate the impact of the employee’s perception of job insecurity, and how it affects the breach of psychological contract, and finally, (c) those who have extended the explanations of the relationship between the rupture and the result, focusing on the analysis of psychological contracts as dynamic processes.

### Organizational Commitment

There are various definitions of organizational commitment one been as the desire on the part of the employee to make high efforts for the good of the institution, longing to remain in it and accept its main objectives and values (Porter and Lawer, 1965). Another widely accepted definition is that of Greenberg and Baron (2008) who define organizational commitment as the degree to which employees identify with the organization where they work, the degree of commitment they show and whether they are willing to leave it. In research related to organizational engagement, three different perspectives can be distinguished. The first one is born from the perspective of social exchange, where the commitment of the individual to the organization is the result of the small investments that he or she has made over time and that would stop his or her voluntary disengagement from the organization (Becker, 1960). This perspective was later developed by Meyer and Allen (1991, 1997) where it was called Commitment to Continuity (CC). The second model, Affective Commitment (CA), leans toward a psychological perspective, where emphasis is placed on the binding force between the person and the organization. It is characterized by the employee’s desire to remain a member of the organization, accepting values and goals from the organization in exchange for certain psychological rewards, such as support or recognition (Mowday et al., 1979; Mathieu and Zajac, 1990). The third perspective, or Normative Commitment (NC) developed by Meyer and Allen (1991) is focuses on the work ethic and the responsibility that the worker acquires, which drives him/her to do his/her job well in any circumstance. This normative commitment has been the source of multiple interpretations regarding its independence as an element of study (Varona, 1993; Ko et al., 1997; Bergman, 2006; González and Guillén, 2008). On the other hand, organizations have assimilated that employees represent their most important asset (Glen, 2006; Fulmer and Ployhart, 2014; Millar et al., 2017). Job satisfaction and job motivation, among other factors, become key aspects for the company’s success. Several attempts have been made at an integrated theory for analyzing motivation at work, covering most approaches and factors involved in employee motivation and expectations (Donovan, 2001; Locke and Latham, 2004), although no complete consensus has been reached. As for organizational commitment, it is closely linked to job satisfaction. This satisfaction depends on many factors, but most are related to what the organization brings to the employee. Some studies indicate that job satisfaction precedes the level of organizational commitment (Meyer et al., 2002; Morrow, 2011), in contrast, other research defends the idea that it is organizational commitment that is a predecessor to job satisfaction (Price and Mueller, 1981; Curry et al., 1986). Organizational engagement of employees has been addressed in remarkable research where one can distinguish between so-called individual theories and process theories. Individual theories are based on the individual, the needs of the individual, and his or her motivation to act in one way or another. In this section we could cite: the Theory of Motivation (Maslow, 1943); the Theory of Hierarchy (Alderfer, 1969); or the Theory of Motivation-Hygiene (Herzberg, 2005). The process theories also include the characteristics of the job or work environment, where other factors surrounding the individual are taken into account and are focused on analyzing why people have different behaviors in relation to the commitment to their organization. Among these process theories are, The Theory of Work and Motivation (Vroom, 1964), Goal Setting Theory (Locke, 1968) and the Theory of Equity (Adams, 1963) (see Culibrk et al., 2018).

### Psychological Contract and Organizational Commitment

At present, there is no generally accepted categorization that encompasses the connection between psychological contract and organizational commitment. However, the literature has strongly related the link between both theoretical constructs, analyzing it from different perspectives, and giving it special relevance in terms of its influence in the field of organizational behavior (Meyer and Allen, 1997; Bunderson, 2001; Lester et al., 2002; Sels et al., 2004; McInnis et al., 2009; Meyer and Parfyonova, 2010; Coyle-Shapiro et al., 2019). Organizational commitment, within the organizational approach, is among the most analyzed consequences in the literature, when explaining the results of the breach of psychological contracts, as opposed to other approaches such as factors oriented to internal third parties (supervisors or co-workers), external third parties (unions or clients) or health (Coyle-Shapiro et al., 2019). On the other hand, research has been carried out that seeks to analyze the relationship of the psychological contract and its variants (relational and transactional psychological contract) with the different types of organizational commitment. In this sense, Meyer and Parfyonova (2010), in their attempt to explain the differentiation between CA and NC, and to reinforce the conceptualization of the latter, point out the importance of this distinction to better understand the processes by which the psychological contract influences employee behaviors. Meyer and Allen (1997); Bunderson (2001), Lester et al. (2002), Sels et al. (2004), and McInnis et al. (2009) point out that psychological contracts have been more related to the CA, however, it is more relevant to relate it to the NC. In addition, Meyer and Parfyonova (2010), suggest that the conception of the type of contract in which the employee is (transactional or relational contract) may have implications on the way in which the employee experiences CK. In this line, they highlight that it is likely that employees with a relational contract experience a sense of moral duty, aligning themselves with the interests of the organization, even at the cost of sacrificing their own objectives. However, employees with a transactional contract, experience their obligation within the reciprocity of a social exchange relationship, consequently, they can respond out of necessity instead of desire and restrict their contributions to what is strictly necessary.

Another connection of the psychological contract with organizational commitment has to do with the most recent conceptualization of the psychological contract, which goes beyond the theory of social interchange; the so-called ideologically charged psychological contracts. This concept is based on the proposal of Thompson and Bunderson (2003), which has been supported by many other authors such as Bingham (2005); El Bedoui et al. (2011) or Vantilborgh et al. (2014), in relation to employee behavior inspired by ideology. This approach suggests that the employee recognizes his or her contribution to the achievement of a greater good aligned with the company’s objectives, for which he or she would be willing to make some sacrifices. In this sense, the employee understands that the achievement itself provides him/her with an intrinsic motivation and this encourages him/her to continue cooperating with the organization in the belief that it is the right thing to do. Under this prism, the behavior that exceeds the psychological contract based on social exchange, is explained from the conception of the commitment with moral objectives that can promote satisfaction, even in the absence of an economic relationship (Shamir, 1990; Aguilera et al., 2007). In this line, Meyer and Parfyonova (2010) try to relate the ideological psychological contract with the affective and normative commitment when; (a) there are ethical foundations, (b) they are transformative and charismatic leaders, or (c) they are employed with collectivist cultures.

### Justification, Objectives, and Practical Implications

It is noted that there is a strong link in the literature between psychological contract and organizational commitment. It is also confirmed that new lines of research continue to appear, such as that which analyzes the relationship between the typologies of psychological contracts and the different types of organizational commitment. Hence, there is an incipient interest in ideologically charged psychological contracts and their close relationship with the CA/NC, -some authors suggest that the investigation of ideologically charged psychological contracts is in an initial phase and invite further research (Coyle-Shapiro et al., 2019). For this reason, a broader approach is necessary, with the purpose of knowing more about the literature around these two great theoretical axes, which explain to a great extent the organizational behavior and the consequences that derive from them. Therefore, the objective of this work is to give visibility to scientific results that provide knowledge of the nexus between psychological contract and organizational commitment, putting the focus on explaining the relationship between them and with other theoretical elements within the area of human resources, as well as identifying the spaces of research that remain to be explored. Hence, in its practical implication, this research can serve business organizations to better focus decision-making on the management of organizational behavior, in an attempt to find greater efficiency and sustainability of human capital. To achieve this objective, this work carries out a review of the research literature that analyzes these two theoretical constructs together. To this end, bibliometric analysis techniques will be used to present a detailed study of the evolution of research and authors who have addressed this issue. The adopted approaches and the incorporation of numerous empirical studies have provided a remarkable sustained literary wealth, which goes from the first works published in the nineties of the past century, to the proliferation of publications that take place in the beginning of the twenty first century.

The Web of Science (WoS) database has been used as a source in the search of scientific publications and SciMat as software for the generation and visualization of maps. A total of 220 articles related to the topic at hand have been used as a starting point. The analysis carried out is divided into two stages; firstly, an attempt to evaluate from a quantitative and qualitative orientation the production by countries, authors and journals, and secondly by means of longitudinal and strategic maps, which show the intensity of the relationships between the topics and their evolution in time, therefore giving an idea of which are the most developed and where future research can be directed.

## Materials and Methods

This work aims to know the trends in the scientific production of *psychological contract* and *organizational commitment*. This research is based on a bibliometric analysis of the scientific literature. A bibliometric analysis examines bibliographic material from an objective and quantitative perspective that is useful for organizing information within a field specification (Albort-Morant and Ribeiro-Soriano, 2016); Therefore, a metric analysis of the bibliography allows us to analyze the details of the main research topics within a domain and the relationships at the micro level, generating useful information for researchers who evaluate scientific activity (Chen and Xiao, 2016; Rey-Martí et al., 2016).

In order to carry out our analysis, two clearly differentiated sets will be described in detail; relationship indicators and activity indicators. The review of the articles in our sample will reveal the evolution of the research related to psychological contract and organizational commitment. Other data that is analyzed correspond to the year of publication of the manuscripts, authors, place of origin, number of citations, impact indices and other characteristics related to scientific production. This approach will provide researchers with a complete vision of what scientific production in this field has meant.

The database used to extract the sample has been Web of Science, where the search was carried out in February 2019. To meet the objectives set out in this research, the location of articles in this database was configured with the following expressions: *“psychological contract”* and *“organizational commitment.”* The year of publication was not limited and was restricted to the Science Citation Index Expanded (SCI-EXPANDED) and Social Sciences Citation Index (SSCI). Thus, those documents whose title, abstract or keywords include the expressions “psychological contract” and “organizational commitment” are extracted. To further refine the search for the selected items, the search is restricted to the categories *“Management,” “Phychology applied,” “Business,”* and *“Sociology.”* This configuration provided a total of 220 items in this source. Usually, in reviews that use bibliometric techniques, those articles that have received a greater number of citations are selected. In this review, all articles that meet the above parameters are included, so that those manuscripts that have not yet reached their highest impact rate are not excluded.

In a first step for a global perspective, we will carry out a descriptive statistical analysis, where the indicators of activity in the literature are detailed. In a second step, where the temporal evolution of these topics will be appreciated, we will make longitudinal, strategic and thematic network maps. In this way, those topics that have had a greater transcendence throughout time will flourish, as well as those that have a lesser current impact, or those that are currently positioning themselves as topics of greater interest in relation to our purpose.

### Software

SciMAT has been used for the construction of longitudinal maps, strategic maps and thematic networks. This allows us to see chronologically what the thematic evolution of the literature under study has been. This software has been developed by SECABA, a research group from the University of Granada, Spain (Cobo et al., 2012).

The following configuration of SciMAT has been carried out for the analysis: the author’s keywords and those coming from the source represent the thematic unit. The equivalence index acts as a measure of similarity to normalize the networks, as well as to create the scientific map of topics and the networks that compose it. The single center clustering algorithm was used.

**FIGURE 1:**
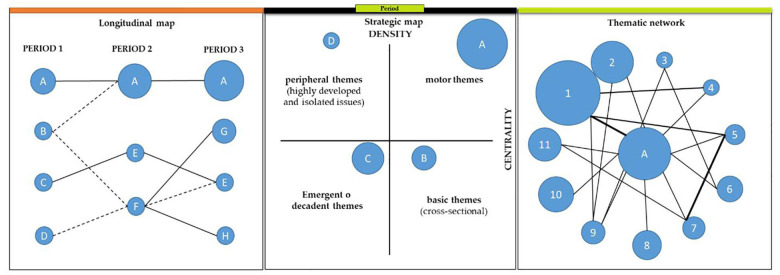
Example of longitudinal map, strategic map and thematic network. Prepared by the authors base in Cobo (2011).

In order to more clearly expose the focus of this research, Figure 1 shows schematically the procedure that has been performed. The longitudinal map (Figure 1, Left) indicates the evolution of literature associated to a particular theme. In this map each period represents by means of spheres the main themes concentrated in a certain number of articles of that period. The evolution from period to period is reflected by connecting lines between the represented items.

A strategic diagram is divided into four quadrants (Figure 1, Center). Motor themes are in the upper right quadrant, peripheral themes in the upper left, emerging themes are in the lower left quadrant, and basic themes are in the lower right quadrant (Cobo et al., 2012). With respect to the first (Figure 1, Center Circle “A”), they indicate that subjects are the motors of the specialty, they have a high density and pronounced centrality, they are very developed and important subjects for the composition of the scientific field, they also have notable relations with concepts applicable to other subjects. The circle “D” (Figure 1, Center), identifies the position that the peripheral themes would occupy, although their importance in the scientific field being analyzed is not very high, if they have very developed internal links and are characterized by being very specialized. The circle “C” (Figure 1, Center) indicates the position that emerging or decadent themes would occupy. Both their density and their centrality are very low, so their development has not been very notable, although they may evolve toward more transcendental themes in the future. The basic themes are represented by the circle “D” (Figure 1, Center). Although they are not sufficiently developed, they are transversal and generic, and therefore represent the basic themes of a scientific field. The third dimension in the strategic map is included through the spheres of the themes, where their volume may represent different bibliometric indicators, such as the number of documents associated with a theme, the number of citations received by the documents associated with each theme, or the H-Index of the theme (Cobo, 2011).

Together with the global network of interconnected themes and keywords, a second thematic network is created, based on the documents associated with each theme. Each thematic network (Figure 1, Right) is assigned the documents that share some keywords with the network, being able to consider two types of networks: main documents and secondary documents. A main document will be one that contains at least two keywords of the thematic network, and those documents that have at least one keyword associated to the thematic network will be called secondary document. In this way, both primary and secondary documents can belong to more than one thematic network (Cobo, 2011). The quantitative and qualitative analysis developed in this research has been based on the network of primary documents.

## Results

### Indicators of Activity in the Literature on Psychological Contract and Commitment

The evolution of the number of publications per year has experienced a notable increase. The sample analyzed consists of 220 articles, the first of which appears in 1994, *“Expatriate Managers and the Psychological Contract”* published by Guzzo, RA, Noonan, KA and Elron, E. in *Journal of Applied Psychology*. This journal is one of the pioneers and remains one of the most productive in this field, although its contribution does not keep a constant pace.

Up to and including the year 2000, the number of investigations published within the chosen sample was only 14 units (6%). Between 2001 and 2010 this number rises to 88 articles, and from 2011 to the present a total of 118 works have been published in these sources. The years between 2005 and 2015 are shown as the most productive with a total of 149 (68%) articles with respect to this sample. Bearing in mind that the current decade (2011–2020) still has a few months of production to go and that the articles cited gain prominence in the years following their publication, it is worth noting that there has been a considerable increase in the pace of publication on this subject.

**FIGURE 2:**
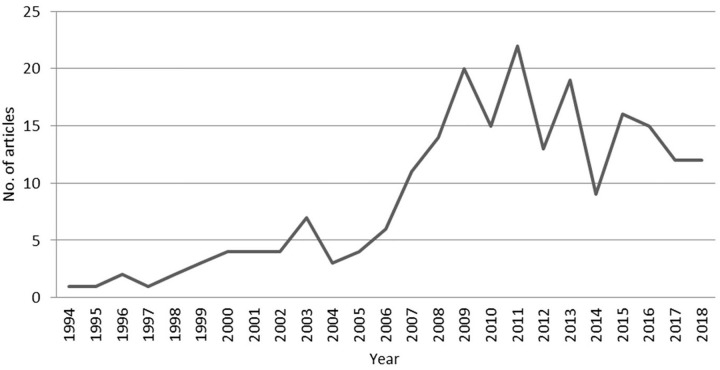
Number of articles by year of publication. Prepared by the authors on the basis of WoS data.

The Figure 2 shows how from 2005 onwards the growth of publications is very high, maintaining an average rhythm of 17 articles per year until 2013, where a pronounced fall can be seen. Until 2005, researchers from the United States of America published on this subject in a significant way with 31 articles, the American country has the largest representation with a total of 58 articles between 1994 and 2018. From 2006 to 2018, researchers from England (24), Holland (16), Australia (15), Canada (15), China (14), and Germany (11) have more than 10 publications. The rest of the documents are distributed among other countries that have not reached 10 in the last 15 years; Spain (5), Taiwan (5), France (4), Greece (4) or India (4) are the most numerous examples. In this phase before the turn of the century, the research carried out by Guzzo, Noonan and Elron entitled *“Expatriate managers and the psychological contract”* (1994) stands out and becomes a reference for later works.

The authors who have published the greatest number of articles in the database consulted are shown in Table 1. The Hirsch Index, or H-index, of each author is also added, which relates the number of publications and citations received.

**TABLE 1:** Authors who have published three or more articles on psychological contract and organizational commitment (1994–2018).

Author	Number of articles	Number of articles as first author	Year of publication last article	H-index in WoS
De Witte, H	10	1	2016	39
De Cuyper, N	9	6	2016	28
Bal, PM	7	4	2016	17
Ng, TWH	6	6	2015	33
Feldman, DC	6	0	2012	52
Schalk, R	5	2	2011	20
Conway, N	4	2	2011	18
Lange, AH	4	0	2015	22
Hofmans, J	4	1	2016	17
Solinger, ON	4	3	2016	5
Billings-Harris, L	3	0	2012	5
Buttner, EH	3	3	2012	10
Guest, D	3	1	2011	30
Jansen, PGW	3	0	2013	13
Lowe, KB	3	0	2012	13
Peiro, JM	3	0	2010	10
Rigotti, T	3	1	2013	11
Vandenberghe, C	3	0	2013	22
Chambel, MJ	3	1	2018	12
Guerrero, S	3	3	2014	6
Parzefall, MR	3	3	2012	7

*Prepared by the authors on the basis of WoS data.*

It is necessary to comment as the author with more publications (10), De Witte, H, only appears as first author in an article of the ten where he participates. On the other hand, De Cuyper, Bal, Ng, and others, are authors who usually appear as the first researcher in the articles in which they participate. The most cited authors per article are not represented in the above list, as their production in this field has not been as numerous. The most cited article of all those analyzed is by Zhao, Wayne, Glibkowski and Bravo who in 2007 published *“The impact of psychological contract breach on work-related outcomes: A meta-analysis” with* 517 citations from other researchers. Another article to be highlighted is the manuscript published by Coyle-Shapiro et al. (2019), *“Psychological Contracts: Past, Present and Future,”* which despite its novelty already has more than 37 citations from other authors and represents a broad systematic review on this subject.

With respect to the journals with the greatest presence in research on Psychological Contract and Organizational Commitment, it can be indicated that the set of 220 articles studied in this work from 1994 to 2018 are distributed in 82 different publications, highlighting the most representative with 19 and 17 research edited. As with research production in this field, the most active journals have appeared in this field since 2005, almost continuously publishing a significant number of articles each year.

The journals with the greatest presence of this sample and its impact factor are indicated in Table 2. Most of them are located in the first or second quartile (Q1–Q2–Q3–Q4) of this theme according to JCR Category. In the cases where a publication is cataloged in more than one category, the one belonging to the area of knowledge according to this research has been chosen.

**TABLE 2:** Journals on psychological contract and organizational commitment.

No.	Journal	Q1	Q2	Q3	Q4	Total items	%
1	International Journal of Human Resource Management		X			19	8.6%
2	Journal of Vocational Behavior	X				17	7.7%
3	Journal of Organizational Behavior	X				9	4.1%
4	Personnel Review			X		8	3.6%
5	Journal of Managerial Psychology			X		8	3.6%
6	Human Relations	X				7	3.2%
7	Journal of Business Ethics	X				6	2.7%
8	Journal of Business Research		X			6	2.7%
9	Journal of Occupational and Organizational Psychology	X				6	2.7%
10	Economic and Industrial Democracy			X		5	2.3%
11	Journal of Applied Psychology	X				5	2.3%
12	Career Development International		X			5	2.3%
13	European Journal of Work And Organizational Psychology	X				4	1.8%
14	Group and Organization Management	X				4	1.8%
15	Journal of Business And Psychology	X				4	1.8%
16	African Journal of Business Management			X		4	1.8%
17	Employee Relations			X		4	1.8%
18	International Journal of Manpower				X	4	1.8%
19	Relations Industrielles-Industrial Relations				X	4	1.8%

*Prepared by the authors on the basis of WoS data.*

The International *Journal of Human Resource Management*, volume 1 of which appeared in 1990, is the most active in this field, with 19 articles since 2003, and continuous production to date. The second publication with 17 articles, *Journal of Vocational Behavior* appears in 1971, and like the previous one from the beginning of the twenty first century it gathers a series of researches on the subject we are concerned with, although as it happens in the general computation it shows more activity from 2008 onwards. In Table 3, the 19 journals listed in Table 2, which indicates the number of articles related to *Psychological Contract* and *Organizational Commitment* that have been published each year, are shown.

**TABLE 3:**
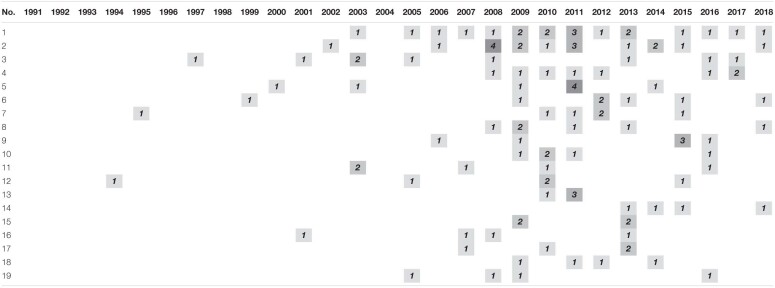
Annual journal activity in articles on psychological contract and organizational commitment. number of articles per year.

*Prepared by the authors on the basis of WoS data. Darker background color means more journal activity in this area.*

As can be seen, except for the *International Journal of Human Resource Management*, no journal represents more than 8% of the published articles, which indicates that few journals have specialized specifically in this matter. It also gives an idea of how this subject is represented from multidisciplinary approaches, which although having limited the search in certain areas of knowledge, the study of the Psychological Contract and the Organizational Commitment admits points of view from various disciplines.

### Content Analysis

#### Evolution of Keywords

In order to analyze the conceptual and thematic evolution of the literature related to organizational commitment and psychological contract, we first analyze how the keywords have evolved, their overlapping, continuity and discontinuity chronologically. For this purpose, 4 periods are established (1994–2004/2005–2009/2010–2014/2015–2018). The criterion of creating blocks with a certain similarity in their amplitude and number of documents has been followed. It was estimated that 5 years periods would favor analysis. For the first block, due to the low level of literary production, it was thought convenient to extend from 5 to 10 years the breadth of the period, even so, it contains the least number of publications with a total of 32 documents. The second and third blocks, both have an amplitude of 5 years and 55 and 78 manuscripts, respectively. In the case of the fourth block, it comprises the last 4 years of the total period under study, and has a total of 55 publications.

**FIGURE 3:**
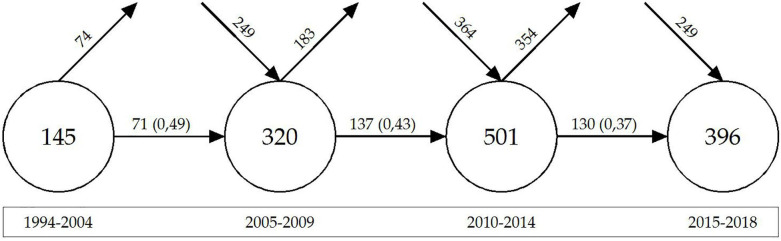
Keywords shared between periods. Prepared by the authors on the basis of SciMAT data.

In the analysis of the evolution of keywords in the area, the methodology of Price and Gürsey (1975) has been used (Figure 3). Each circle represents a chosen period, the figure inside it indicating the number of different keywords in that period. The horizontal arrows that join the circles represent the keywords shared that pass from one period to the next, so they remain between the two periods. The figure in parentheses indicates the stability index or overlapping fraction (between period 1 and 2, the stability index was 49%, which decreased between period 2 and 3, and between period 3 and 4. This indicates the growth of keywords that have been associated with this theme. This indicates the growth of keywords that have been associated to this theme. An arrow appears at the top of each period to inform us of how many keywords no longer appear in the following period. At the opposite point, the arrow that enters diagonally in the circle indicates the number of new keywords that have been incorporated during this period. As you can see in Figure 3, in each period new keywords are added and others are no longer used with respect to adjacent periods. A large number of keywords is observed, which gives an idea of the diversity of topics adjacent to those dealt with by the central core object of this study. As for the analysis of periods, the number of keywords is closely related to the number of publications, so the third (2010–2014) is where more keywords have coexisted with a total of 501. The stability index has remained between 0.49 and 0.37 which, although decreasing, shows a high strengthening of the vocabulary by the scientific community when describing the published documents (Cobo et al., 2012).

#### Longitudinal Analysis of the Theme

**FIGURE 4:**
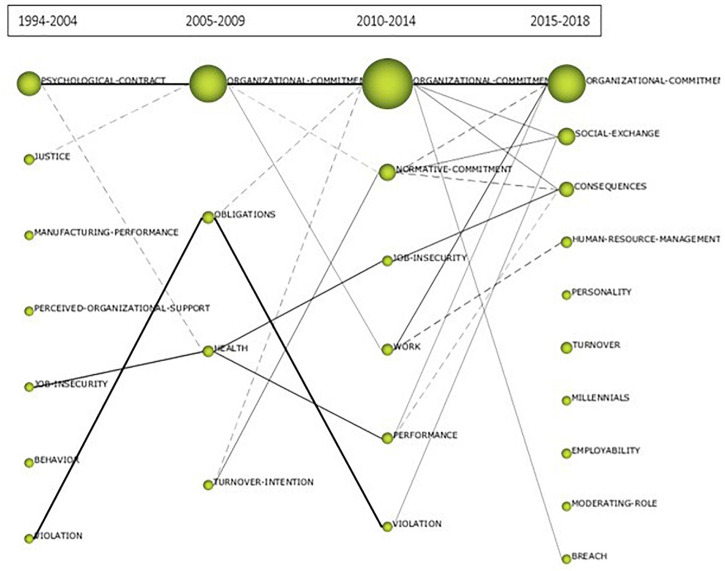
Evolution of the theme of organizational commitment and psychological contract. primary documents. Prepared by the authors on the basis of SciMAT data.

Once the evolution of keywords has been analyzed, the study would focus on how the subject has evolved. In order to do this, we rely on Figure 4, where the relations of the themes are represented by periods according to the volume of primary documents. In the case of Psychological Contract and Organizational Commitment, there is continuity in each of the periods. In the first period, the subject revolves more around the Psychological Contract, giving way to Organizational Commitment in the following periods. However, in the central blocks (2005–2009 and 2010–2014), the issues are closely related. In the case of the subject that has to do with the violation of the psychological contract, it evolves in all periods toward the conception of obligations and toward social exchange in the last period. Job insecurity remains in force in all periods, resulting in the second with health and in the last two with performance or social exchange. In the last period (2014–2018) there are themes that are not related to those of previous periods, such as *personality*, *turnover*, *millennials, employability* or *moderating-role* and that, due to their density, are themes with certain possibilities of becoming driving themes in the near future.

#### Thematic Analysis Through Strategic Diagrams

##### Period 1994–2004

**FIGURE 5:**
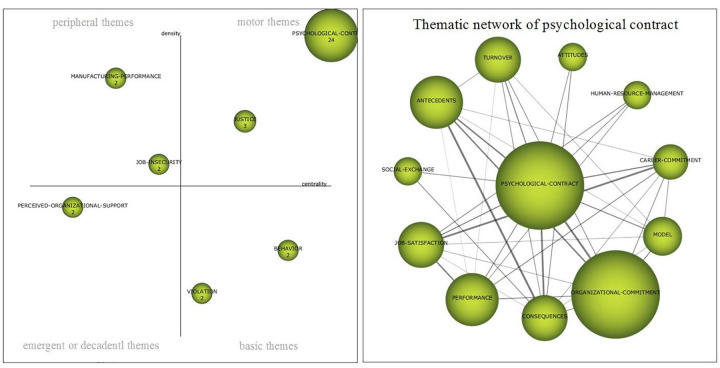
Strategic map 1994–2004. Prepared by the authors on the basis of SciMAT data.

The strategic diagram in Figure 5 represents an image of the situation of the theme in the first decade in which the central themes of this study, Psychological Contract and Organizational Commitment, began to be jointly researched. The main driving themes are *psychological contract* and *justice*. The first one has a high level of centrality and density (Table 4) which makes it the main asset of the subject, at the same time it stands out from the others, both in the quantitative plane with 28 documents, and in the qualitative plane with an H-index of 24, in the case of the second, *justice*, with an H-index of 3, it only has 4 documents related to satisfaction, the behavior of citizens or trust. As basic topics are those related to *behavior* or *violation* of the psychological contract. As for emerging or decadent issues, there is *organizational support*, which has a high density, although the centrality is relatively low. In the upper left quadrant, in which peripheral themes are visualized, it is represented by *performance* or *job insecurity*, both considerably developed, although with little production and low H-index.

**TABLE 4:** Cluster period 1994–2004.

Name	Centrality	Centrality range	Density	Density range	Documents	H-index
Psychological-contract	99.82	1.00	58.11	1.00	28	24
Justice	13.62	0.71	37.50	0.71	4	3
Manufacturing-performance	2.00	0.29	50.00	0.86	2	2
Perceived-organizational-support	1.67	0.14	33.33	0.43	2	2
Job-insecurity	3.17	0.43	33.33	0.57	2	2
Behavior	15.12	0.86	25.00	0.29	2	2
Violation	5.04	0.57	16.67	0.14	2	2

*Prepared by the authors on the basis of WoS data.*

With the intention of knowing which literary activity makes up the main driving theme, which in this first period is a *psychological contract*, it would be interesting to analyze its thematic network (Figure 5). The study of psychological contracts is intimately related to *organizational commitment*, but it is also closely related to the analysis of *performance*, *job satisfaction*, *rotation*, *antecedents* and the *consequences* of the same (both topics that in turn maintain an important intensity in their relationships), as well as with other topics with less intensity such as *professional commitment, models, human resources management or social exchange*.

The five publications with the greatest impact of this network between 1994 and 2004 were, (i) Whitener (2001), *“High Commitment” Human Resource Practices Affect Employee Commitment?* (times cited: 443); (ii) Raja et al. (2004), *The Impact Of Personality On Psychological Contracts* (times cited: 308); (iii) Guzzo et al. (1994), *Expatriate Managers And The Psychological Contract* (times cited: 304); (iv) Scandura and Lankau (1997), *Relationships Of Gender, Family Responsibility And Flexible Work Hours To Organizational Commitment And Job Satisfaction* (times cited: 236), and (v); Meyer and Smith (2000), *Hrm Practices And Organizational Commitment: Test Of A Mediation Model* (times cited: 210).

##### Period 2005–2009

**FIGURE 6:**
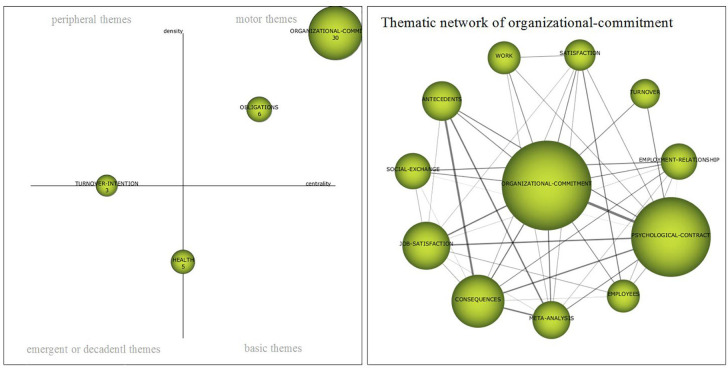
Strategic map 2005–2009. Prepared by the authors on the basis of SciMAT data.

In the second block (2005–2009), in the association Psychological Contract—Organizational Commitment as a driving theme, there is a change of leadership (Figure 6). The number of documents and the quality (H-index) increased in this second period, from 28 to 51 and from 24 to 30, respectively (Tables 4, 5). As the second driving theme, *obligations* appear, with 7 publications related to perceptions, violation and the rupture of the psychological contract. In this period there is a peculiarity in which two clusters are generated on the topics *health* and *turnover-intention.* The first is at the border of the quadrant of emerging themes with the basic themes that, as has been commented (Figure 4), was an evolution—from the previous period—of the *job-insecurity* theme and evolved, in the following period, toward themes such as *job-insecurity* or *performance.* In the case of the latter, it lies between the quadrant of emerging or decadent themes and peripheral themes, as seen earlier created in this period, but evolves in the following one toward *normative-commitment.* Both topics have few documents, between 5 and 3, respectively, and in the qualitative aspect an H-index of 5 and 3 equally (Table 5).

**TABLE 5:** Cluster period 2005–2009.

Name	Centrality	Centrality range	Density	Density range	Documents	H-index
Organizational-commitment	118.27	1.00	57.38	1.00	51	30
Obligations	27.46	0.75	34.80	0.75	7	6
Health	16.02	0.50	13.33	0.25	5	5
Turnover-intention	3.30	0.25	16.07	0.50	3	3

*Prepared by the authors on the basis of WoS data.*

The thematic network of *organizational commitment*, for this period, as main driving theme (Figure 6) is constituted, in the first place, by *psychological contract* and closely, by *antecedents* and *consequences*—al as well as in period anterior—, and *job-satisfaction*. On the other hand, there are two issues that are closely related to organizational engagement, such *as social-exchange* and *employment-relationship.* During this period there is a great deal of interest in knowing about the literature on this subject, with 12 publications on the subject of *meta-analysis.*

With respect to the five publications with the greatest impact between 2005 and 2009, the following can be found; (i) Zhao et al. (2007), *The Impact Of Psychological Contract Breach On Work-related Outcomes: A Meta-analysis* (times cited: 538); (ii) Cheng and Chan (2008) *Who Suffers More From Job Insecurity? A Meta-analytic Review* (times cited: 335); (iii) Bentein et al. (2005), *The Role Of Change In The Relationship Between Commitment And Turnover: A Latent Growth Modeling Approach* (times cited: 198); (iv) De Cuyper and De Witte (2006), *The Impact Of Job Insecurity And Contract Type On Attitudes, Well-being And Behavioural Reports: A Psychological Contract Perspective* (times cited: 183); and (v) Bal et al., 2008), *Psychological Contract Breach And Job Attitudes: A Meta-analysis Of Age As A Moderator* (times cited: 160).

##### Period 2010–2014

**FIGURE 7:**
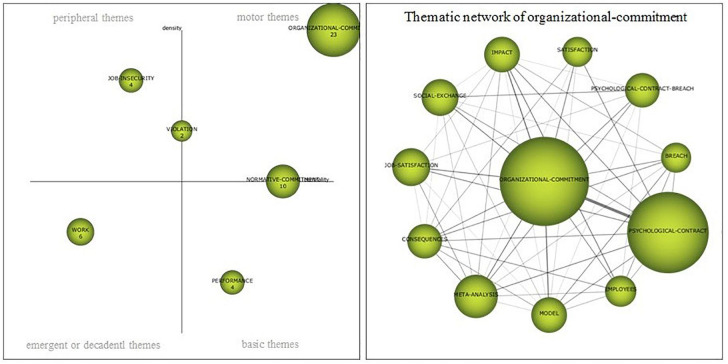
Strategic map 2010–2014. Prepared by the authors on the basis of SciMAT data.

The third block (2010–2014) continues its *organizational commitment* as the main driving theme (Figure 7), which although it is present in more publications, 74 as opposed to 51 in the previous period, from a qualitative point of view has worsened, going from a 30 to a 23 H-index (Tables 5, 6). At the frontiers of the driving themes there are two themes that could belong to this classification. The first is in the line that divides the peripheral themes of the emerging or decadent, *normative-commitment*^1^, which is an evolution, as we could see, of *turnover-intention* and that has experienced a greater centrality between its publications—goes from 3.3 to 26.83—, giving rise to the potential necessary to become a driving theme, with a notable increase in its H-index, which goes from 3 to 10. The second, *violation*, is at the frontier of peripheral issues. In this period it is again configured as a driving theme with *violation*, but with a lower density and centrality and, in quantitative and qualitative terms, fewer documents and lower H-index. In the basic and peripheral themes, there are *job-insecurity* and *performance*, themes that have evolved from *health* of the previous period, to transform the first into a peripheral theme, with a notable density and centrality and the second into a basic theme, little developed, with low centrality and density, however both have the same H-index (4). As an emergent or decadent theme appears *work*, which with an H-index of 6, has an acceptable density, but a very low centrality (Table 6).

**TABLE 6:** Cluster period 2010–2014.

Name	Centrality	Centrality range	Density	Density range	Documents	H-index
Organizational-commitment	100.48	1.00	53.64	1.00	74	23
Normative-commitment	26.83	0.83	14.76	0.50	15	10
Job-insecurity	7.85	0.33	17.50	0.83	4	4
Work	7.13	0.17	12.19	0.33	6	6
Performance	11.30	0.67	8.33	0.17	5	4
Violation	10.73	0.50	16.07	0.67	3	2

*Prepared by the authors on the basis of WoS data.*

In the period 2010–2014, the thematic network of *organizational commitment* (Figure 7), as the main driving theme, has its densest relationship with *psychological contract*, followed by *meta-analysis*, which is again present with some consistency, on the other hand, other themes such as *job-satisfaction, consequences, psychological-contract-breach* or *social-exchange* are related to each other and strongly related to organizational commitment.

The five publications with the greatest impact between 2010 and 2014 were; (i) Ng et al. (2010) *Psychological Contract Breaches, Organizational Commitment, And Innovation-related Behaviors: A Latent Growth Modeling Approach* (times cited: 94); (ii) Tremblay et al. (2010), *The Role Of Hrm Practices, Procedural Justice, Organizational Support And Trust In Organizational Commitment And In-role And Extra-role Performance*, (times cited: 83); (iii) Direnzo and Greenhaus (2011), *Job Search And Voluntary Turnover In A Boundaryless World: A Control Theory Perspective* (times cited: 82); (iv) Lub et al. (2012); *Different Or Alike? Exploring The Psychological Contract And Commitment Of Different Generations Of Hospitality Workers* (times cited: 79); and (v) Deconinck (2011), *The Effects Of Ethical Climate On Organizational Identification, Supervisory Trust, And Turnover Among Salespeople* (times cited: 69).

##### Period 2015–2018

**FIGURE 8:**
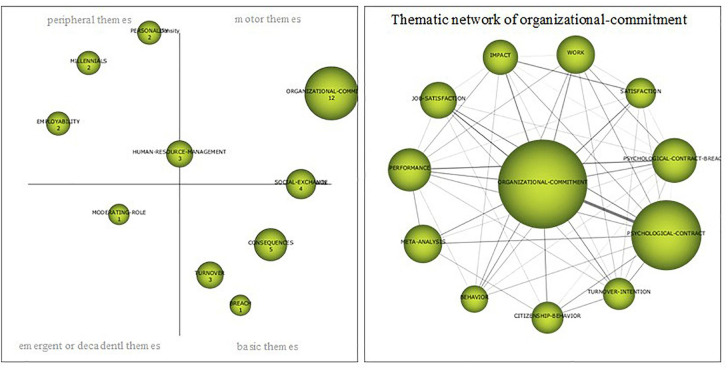
Strategic map 2015–2018. Prepared by the authors on the basis of SciMAT data.

The last period (2015–2018) is characterized by the proliferation of themes, fundamentally in publications classified as peripheral and basic and as a consequence, by a lower density in the works related to the main driving theme, *organizational commitment* (Figure 8). As in the previous period, there are motor themes that are right in the line that divides this quadrant with the basic themes or with the peripheral themes. At the border with the basic issues is a *social-exchange*, which is an evolution of *normative-commitment*, a theme inherited from the previous period, which retains virtually its range of density and centrality. With respect to its thematic network, it is interesting to highlight that, due to its evolution, it constitutes a line of research that seems to be consolidating, in addition to the normative commitment, toward issues related to the affective-organizational commitment and the importance of the perception of the organizational support, the role of the leader or the organizational trust, the professional commitment or the behavior of the citizenship. On the other hand, *human-resource-management* appears for the first time and does it as a *work* evolution,—in the previous period it was considered as a emerging topic*—*with topics about the management and retention of talent. With respect to the basic issues, *consequences*, *turnover* and *breach* appear, the first comes from the previous period as an evolution of *job-insecurity*, located among the peripheral issues. In the quadrant of the basic themes there are three clusters that appear for the first time and that have no link with themes in previous periods; *personality*, *millennials* and *employability*, have a considerable density and could become future driving themes that point to lines of research related to work attitudes, self-esteem, organizational commitment and the perspective of the psychological contract in the new generations, the exchange of knowledge or work opportunities. Finally, with respect to emerging issues (Table 7), *moderating-role* appears for the first time, with publications analyzing the importance of organizational support in job insecurity and in performance or the role of cultural values in the psychological contract.

**TABLE 7:** Cluster period 2015–2018.

Name	Centrality	Centrality range	Density	Density range	Documents	H-Index
Organizational-commitment	210.53	1.0	45.66	0.8	52	12
Social-exchange	103.86	0.9	21.49	0.5	16	4
Consequences	81.13	0.8	15.43	0.3	12	5
Human-resource-management	24.01	0.5	34.44	0.6	5	3
Personality	22.89	0.4	125.00	1.0	2	2
Turnover	24.71	0.6	13.95	0.2	6	3
Millennials	5.91	0.2	77.78	0.9	2	2
Employability	4.35	0.1	37.04	0.7	3	2
Moderating-role	7.19	0.3	16.67	0.4	2	1
Breach	27.38	0.7	8.33	0.1	2	1

*Prepared by the authors on the basis of WoS data.*

In the thematic network of *organizational-commitment* as the main driving theme (Figure 8), it maintains the most intense relationship with the *psychological-contract*, on the other hand, there is an important connection between both themes and *job-satisfaction.* Other subjects among which it is related with certain intensity are *psychological-contract-breach, performance, work, citizenship-behavior, turnover-intention* or *meta-analysis*—than also appears in this period.

The five publications with the most impact between 2015 and 2018 are; (i) Deery and Jago (2015), *Revisiting Talent Management, Work-life Balance And Retention Strategies*, (times cited: 45); (ii) Ng (2015), *The Incremental Validity Of Organizational Commitment, Organizational Trust, And Organizational Identification*, (times cited: 29); (iii) Choi et al. (2015), *Understanding Organizational Commitment: A Meta-analytic Examination Of The Roles Of The Five-factor Model Of Personality And Culture*, (times cited: 27); (iv) Vander Elst et al. (2016), *Perceived Control And Psychological Contract Breach As Explanations Of The Relationships Between Job Insecurity, Job Strain And Coping Reactions: Towards A Theoretical Integration*, (times cited: 24); (v) Solinger et al. (2016), *Bouncing Back From Psychological Contract Breach: How Commitment Recovers Over Time*, (times cited: 23).

## Discussion

We start from the idea that this work does not intend to carry out a revision of the literature in a deep and systematic way. The methodology used leads us to make visible, in a longitudinal and relational way, how the topics related to psychological contract and organizational commitment have been developed throughout time, and to detect which topics have been leading the literature. This work provides a new perspective on the nexus of these two theoretical constructs related to organizational behavior and sheds light on the issues that have occupied more central positions and which have had a greater density, also providing information on the levels of quality of research (h-index), authors and journals that have been interested in the subject and what level of specialization they have had. This methodological approach also makes it possible to know the state of the subject, in terms of the degree of maturity or saturation, where the research is headed and what spaces have not yet been addressed.

With respect to the findings found in the sample analyzed, it is confirmed that there are key aspects within the axis of psychological contract and organizational commitment that the literature has studied extensively, such as normative commitment, social interchange, violation of the psychological contract, job satisfaction, justice, job insecurity, organizational citizen behavior, performance, or the intention to leave work (Porter et al., 1998; Cassar, 2001; Coyle-Shapiro and Kessler, 2002; Topa and Morales, 2005; Betanzos and Paz, 2007; Coyle-Shapiro et al., 2019). However, there are other themes that, although they may have sufficient entity in the research of either construct independently (psychological contract and organizational commitment), have not been developed from the perspective of the relationship between the two, lacking sufficient centrality and density to represent a cluster and, as a consequence, to position itself as a driving theme and set a trend. Therefore, it is considered that they have not yet been developed or are in an embryonic phase. Hence, we find research such as the analysis of psychological contracts with a strong ideological charge and their relationship with the public sector; the analysis of the organizational context in aspects such as the restructuring and reduction of organizations; how factors oriented to internal third parties (supervisors or colleagues) or external third parties (unions or clients) affect them; analysis of how intercultural differences or horizontal-individualist or vertical-collectivist cultures may affect the perception of the breach of psychological contract; or analysis of demographic variables such as employment status, professional category, age, or gender (Rousseau, 1995; Turnley and Feldman, 1999; Costa et al., 2017; Coyle-Shapiro et al., 2019).

## Conclusion

The literature related to psychological contract and organizational commitment has been producing work independently, providing valuable knowledge in order to better address efficiency within the organizational context. The objective of this work was to know more about the literature of these concepts, that is, to give visibility to the lines of research that try to explain, from all possible perspectives and approaches, what effects the psychological contract has on organizational commitment and both on HR management in organizations.

From the results of this work, it can be inferred that there is indeed an important scientific production that relates the concepts of psychological contract and organizational commitment. This begins at the beginning of the nineties of the last century, in an insignificant way and it is not until 2005 when interest in this subject really takes hold. The most fruitful years in terms of the number of works were between 2006 and 2016, where 72% of the entire sample was concentrated. It should be noted that the publications analyzed belong mostly to journals of the first and second quartile of Social Science Citation Index (*SSCI*) and Science Citation Index Expanded *(SCI-EXPANDED)*, which gives the subject a certain level of relevance, however, none of the journals represents more than 10% of published articles, which shows that there is no specialization of the subject by any of them. In terms of production by country, researchers from the United States of America with 58 documents and England with 24 represent more than 37% of the published works.

The results suggest that the basis of research on psychological contract and organizational commitment has been developed, and that, in recent years, issues have emerged that are beginning to consolidate as lines that focus on more current needs or sensitivities. However, there are research trends in psychological contract and organizational engagement that, although they are likely to begin to obtain results independently, in the field of study between the two constructs (see section “Discussion”), they are not relevant.

From the longitudinal perspective, between 1994 and 2004, *psychological contract* is positioned as a topic with greater density and centrality, however, from 2005, *organizational commitment* takes over with strength and distance progressively. At the same time, they have survived over time with different issues such as the sense of justice, the perception of obligations within the framework of the psychological contract and its violation, the normative commitment or the management of human resources. Job insecurity, sometimes as a peripheral issue and sometimes as a basic issue, has been part of the focus of the central theme. With some distance, it has also happened to the study of performance or behavior. In the last period, works oriented toward a more current social demand emerge, such as employability or the study of new generations (millennials and generation-Y) focused on organizational commitment, satisfaction or the retention of talent which, due to their density, are well developed and which could end up being driving themes in the future; however, the development of other themes, which are currently also especially sensitive, such as the management of diversity and gender equality, is missed.

It must be recognized that this work is not without its limitations. The 220 articles selected for this analysis come only from the Web of Science (WoS) database. This fact may unintentionally exclude important contributions that have been made in other sources, however, the results obtained indicate that the sample used has been sufficiently large. Neither have those studies published in non-academic journals or books been included in the analysis carried out, although they have been taken into account in the introduction and approach to the research.

### Future Research

Future publications should be oriented toward the development of works that continue the investigation of topics that have emerged in recent years and that are related to current socio-economic change, such as new generations and the retention of talent, and among other topics. It would also be necessary to delve into certain topics that have not shown a significant presence and that we consider important as sensitive issues, such as the management of diversity or gender equality in relation to the psychological contract and organizational commitment, as well as the development of the psychological contract with a strong ideological charge and its relationship with the normative commitment, or the transversal analysis of how aspects such as certain demographic factors, interculturality or the organizational context affect and/or moderate both theoretical constructs.

## Author Contributions

JH and CHR designed, performed, analyzed the research, wrote the manuscript, searched literature, analyzed, and verified the data of this article. Both authors contributed to the article and approved the submitted version.

## Conflict of Interest

The authors declare that the research was conducted in the absence of any commercial or financial relationships that could be construed as a potential conflict of interest.

## Footnotes

1 The theme that preceded *normative-commitment* in the period 2004–2009 was *turnover-intention, it was* at the border between emerging or decadent themes (lower left quadrant) and peripheral themes (upper left quadrant).
